# Hierarchical Effector Protein Transport by the *Salmonella* Typhimurium SPI-1 Type III Secretion System

**DOI:** 10.1371/journal.pone.0002178

**Published:** 2008-05-14

**Authors:** Brit Winnen, Markus C. Schlumberger, Alexander Sturm, Kaspar Schüpbach, Stefan Siebenmann, Patrick Jenny, Wolf-Dietrich Hardt

**Affiliations:** 1 Institute of Microbiology, ETH Zürich, Zürich, Switzerland; 2 Institute of Fluid Dynamics, ETH Zürich, Zürich, Switzerland; University of Minnesota, United States of America

## Abstract

**Background:**

Type III secretion systems (TTSS) are employed by numerous pathogenic and symbiotic bacteria to inject a cocktail of different “effector proteins” into host cells. These effectors subvert host cell signaling to establish symbiosis or disease.

**Methodology/Principal Findings:**

We have studied the injection of SipA and SptP, two effector proteins of the invasion-associated *Salmonella* type III secretion system (TTSS-1). SipA and SptP trigger different host cell responses. SipA contributes to triggering actin rearrangements and invasion while SptP reverses the actin rearrangements after the invasion has been completed. Nevertheless, SipA and SptP were both pre-formed and stored in the bacterial cytosol before host cell encounter. By time lapse microscopy, we observed that SipA was injected earlier than SptP. Computer modeling revealed that two assumptions were sufficient to explain this injection hierarchy: a large number of SipA and SptP molecules compete for transport via a limiting number of TTSS; and the TTSS recognize SipA more efficiently than SptP.

**Conclusions/Significance:**

This novel mechanism of hierarchical effector protein injection may serve to avoid functional interference between SipA and SptP. An injection hierarchy of this type may be of general importance, allowing bacteria to precisely time the host cell manipulation by type III effectors.

## Introduction

Numerous symbiotic and pathogenic bacteria employ type III secretion systems to manipulate eukaryotic hosts. These TTSS function as molecular syringes injecting bacterial “effector” proteins directly into the cytosol of eukaryotic cells [1] where they manipulate signaling pathways to establish symbiosis or infectious disease.

Efficient host cell manipulation is based on the delivery of a complex mixture of effector proteins. Each effector protein activates or blocks a particular host cell signaling pathway. The prompt manipulation right after the host cell encounter is thought to result from fast effector protein injection [2], [3] and from functional cooperation between the different effector proteins [4]. However, this early phase of the bacteria-host interaction is still poorly understood.

To study the early phase of host cell manipulation in more detail, we analyzed the TTSS-1 encoded on *Salmonella* pathogenicity island 1 (SPI-1) of the enteropathogenic bacterium *Salmonella enterica* serovar Typhimurium (*S*. Typhimurium; [5]). *S*. Typhimurium employs TTSS-1 to inject a mixture of >10 different effector proteins into host cells in order to invade the host's intestinal mucosa [6];[4]. A pool of these effector proteins is pre-formed in the bacterial cytosol and gets injected within a few minutes after the TTSS-1 apparatus is activated by host cell contact [4]. Surprisingly, some effector proteins have opposing functions inside the host cell: SipA, SopB, SopE and SopE2 trigger actin polymerization, membrane ruffling and host cell invasion [7], [8], [9], [10], [11], [12], while SptP disrupts these responses and allows the host cells to regain their normal architecture within 1–2 hours after invasion [13]. If all pre-formed effector proteins were injected at the same time, SptP could potentially cancel out the activity of SipA, SopB, SopE and/or SopE2. We speculated that this could be avoided if SptP was injected later than SipA, SopB, SopE and/or SopE2. Our data show that there is indeed a hierarchy of effector protein injection: SipA and SopE are injected earlier than SptP. This establishes hierarchical effector protein injection as a novel concept in host cell manipulation by TTSS.

## Results

### *sipA*, *sopE* and *sptP* are co-expressed in *S*. Typhimurium

Host cell contact triggers effector protein injection within a few seconds after *S*. Typhimurium has docked to a host cell. The pre-formed SipA- and SopE-pools are delivered within 80–300 seconds [2]. It had remained unclear whether all components of the pre-formed effector protein cocktail were delivered coequally during this early period. We hypothesized that some type of hierarchy may exist. Based on its capacity to reverse *Salmonella*-induced actin rearrangements [13], we speculated that SptP might be injected later than SipA and SopE. This hypothesis was tested by adapting a time lapse microscopy method which we had developed, recently [2]. This method monitors the depletion of intra-bacterial effector protein pools upon TTSS-1 injection (see below).

**Figure 1:**
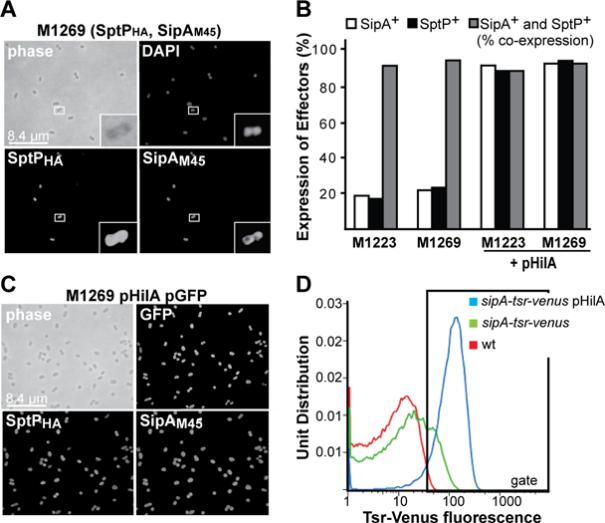
SipA and SptP are co-expressed in *S*. Typhimurium. (A) SipA and SptP expression in wt *S*. Typhimurium. M1269 was grown under TTSS-1 inducing conditions, immobilized on gelatine-coated coverslips, fixed, permeabilized and SptP and SipA pools in the bacterial cytosol were immuno-strained (Materials and Methods). DNA was stained with DAPI. (B) Fraction of bacteria harboring SipA (open bars), SptP (black bars) and fraction of the SipA^+^ bacteria also harboring SptP (grey bars); Data was obtained from experiments as described in (A) and (C); n>400 bacteria, two independent experiments; (C) SipA and SptP expression in *hilA* over-expressing *S*. Typhimurium. M1269 (pHilA) was grown and analyzed as described in (A). (D) FACS-analysis of pHilA-induced *sipA*-expression. M2001 harbors the transcriptional reporter *tsr-venus* integrated downstream of the chromosomal *sipA* gene. FACS analysis of Tsr-Venus fluorescence is shown for M2001 and M2001 (pHilA). Wt *S*. Typhimurium (w/o tsr-venus reporter) served as a negative control. The gate is indicated as a box harboring 1.1% of wt control bacteria, 19.6% of M2001 and 90.8% of M2001(pHilA).

First, we characterized the bacterial strains optimally suited for our study. For this purpose, we analyzed the intra-bacterial SipA-, SopE- and SptP-pools at the single cell level. Before host cell contact, SipA, SopE and SptP were highly expressed by ∼15–25% of the wild type *S*. Typhimurium population (Fig. 1A and B; data for SopE not shown). The levels were ∼6000 molecules SipA, ∼3000 molecules SopE and ∼10000 molecules SptP (not shown). Generally, bacteria expressing one of the three effector proteins also expressed the other two effector proteins (≥90% co-expression; Fig. 1B). Thus, all bacteria of the “TTSS-1 effector expressing” sub-population had the identical, pre-formed effector protein repertoire. However, the heterogeneity represented a challenge for our effector protein injection assay (see below).

To optimize effector protein expression we over-expressed *hilA*, which encodes a central, positive regulator of TTSS-1 [22]. *hilA* over-expression resulted in *sipA*-, *sopE*- and *sptP*-expression by ≥90% of the *S*. Typhimurium (pHilA) population without compromising host cell invasion (Fig. 1B,C; Supplementary Fig. S1A; data for SopE not shown). Also, the pre-formed effector protein pools in the bacterial cytosol were somewhat elevated in the HilA-over-expressing strains (∼25000 molecules SipA, ∼25000 molecules SopE and ∼25000 molecules SptP per bacterial cell; Supplementary Fig. S1B and data not shown). FACS analysis of *S*. Typhimurium strains carrying a transcriptional reporter (tsr-venus; green fluorescence [21]) for *sipA*-expression confirmed that *hilA* over-expression mediated increased *sipA* expression by individual bacteria and homogeneous *sipA* expression in the entire *S*. Typhimurium population (Fig. 1D). Thus, HilA-over-expression yielded an invasive *S*. Typhimurium population which was homogeneous with respect to their pre-formed TTSS-1 effector protein pools (SipA, SopE and SptP).

Infections of COS7 tissue culture cells with wild-type bacteria verified that the intra-bacterial SipA-, SopE- and SptP- pools were depleted well within 30min after host cell contact (data not shown). Inside the host cell, SipA accumulated in small, well defined foci right at the bacteria-host cell interface [2], while SopE and SptP were spread within the host cell (not shown). Similar observations were made with HilA-over-expressing strains. These data indicated that *S*. Typhimurium and strains over-expressing HilA were well suited for studying SipA-, SopE- and SptP-injection into host cells.

### A transport hierarchy: SipA is injected earlier than SptP

**Figure 2:**
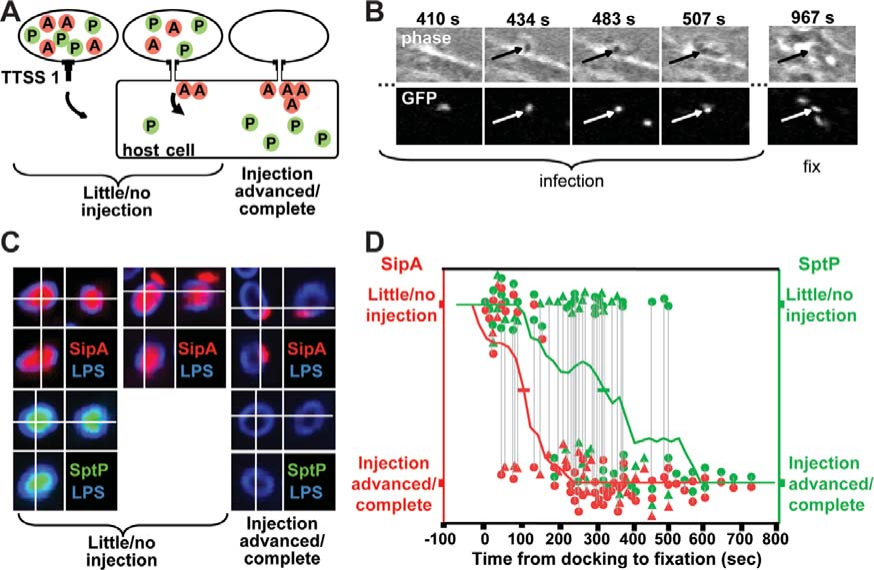
Hierarchical injection of SipA and SptP. (A) Phases of the SipA- and SptP injection process; (B) Typical time lapse movie of the infection process. The bacteria harbored a pGFP plasmid to facilitate detection (GFP fluorescence) in the presence of membrane ruffling (phase contrast). (C) Representative images of M1269 and M1223 (pHilA, pGFP) in the early/intermediate or late phase of SipA and SptP injection into COS7 cells. Cells were fixed, permeabilized with lysozyme, and immunostained for LPS (blue), SipA (red) and SptP (green). (D) Time course of SipA and SptP depletion from *hilA*-overexpressing bacteria during the infection of COS7 cells. Infection was monitored as in (B) and intrabacterial SipA- and SptP pools were stained as in (C). For each bacterium, the graph shows the time between docking and fixation, the presence/absence of SipA (red) and SptP (green) in the bacterial cytosol. Gray lines connect SipA and SptP data from the same bacterium. Circles represent data obtained from M1223 (pHilApGFP; *sipA_HA_sptP_M45_*) and triangles data from M1269 (pHilApGFP; *sipA_M45_sptP_HA_*). The data was fitted using a rolling average algorithm (red and green lines, see Materials and Methods) to determine when injection was completed with 50% probability (t_50%_). tu = time units.

SipA is injected right after the pathogen has docked to a host cell [2]. To test whether SipA- and SptP- are injected in a hierarchical manner, we monitored their depletion from the bacterial cytosol as a function of time (Fig. 2A; see [2]). COS7 cells were infected with GFP-expressing *S*. Typhimurium (pGFP) and in the presence of the antibiotic chloramphenicol (Material and Methods) which inhibits de novo bacterial protein synthesis without affecting TTSS-1 function [23], [24]. Bacterial docking was monitored by time lapse microscopy (phase contrast and GFP-fluorescence) and cells were fixed after 15 min of infection (Fig. 2B). The time-lapse movie revealed the time that each bacterium had spent on the host cell before fixation and the presence/depletion of SipA and SptP in the bacterial cytosol was determined by indirect immuno-fluorescence microscopy using specific antibodies against the epitope-tags present on the effectors (Materials and Methods). Each cell-associated bacterium was assigned to one of the following two categories: (i) Effector protein detectable in the bacterial cytosol (ongoing-, little- or no injection), or (ii) No effector protein detectable in the bacterial cytosol (injection completed; Fig. 2A and C)). In the case of SipA, we could also detect cases with SipA in the bacterial and in the host cell cytosol. These bacteria were assigned to category (i).

First, we analyzed the hierarchy of SipA and SptP injection by *S*. Typhimurium over-expressing HilA (M1223 (pGFPpHilA); *sipA_HA_sptP_M45_*) and M1269 (pGFP pHilA); *sipA_M45_sptP_HA_*). In these strains, ∼90% of the bacterial population harbored a pre-formed effector protein pool and this pool included ∼25000 SipA and ∼25000 SptP molecules (see Fig. 1). Our analysis of effector-depletion from the bacterial cytosol showed that SipA injection was completed significantly earlier than SptP injection (t_50%_(SipA) = 90 to 120 sec vs. t_50%_(SptP) = 315 to 370 sec; p<0.01, see Materials and Methods), Fig. 2D). Identical results were obtained with both strains, M1269 and M1223, which differed only by the nature of the epitope tags used for detecting SipA and SptP (Table 1). This indicated that there is indeed a hierarchy of effector protein injection.

**Table 1:** TTSS-injection times for SipA and SptP in different *S*. Typhimurium strains.

Strain	Eff. 1	Eff. 2	background	pHilA	n*	Delivery time (t_50%_ in sec)†		
Wild type (no pHilA)						SipA	SptP	*p*(t_50%_)‡
M1269	SipA_M45_	SptP_HA_	wt	no	20	75	290	<0.001
**pHilA containing strains**								
M1223	SipA_HA_	SptP_M45_	wt	yes	57	120	370	<<0.001
M1269	SipA_M45_	SptP_HA_	wt	yes	34	90	315	<0.01
M1252	SipA_HA_	SptP_M45_	Δ*sopABEE2*	yes	80	215	595	<<0.001

* number of bacteria analyzed

† determined by rolling average analysis of the data (see Materials and Methods for details)

‡ statistical analysis of data points lying between the t_50%_ values (see Materials and Methods)

The hierarchy of injection was confirmed in two additional experiments. In the first experiment, we employed a *S*. Typhimurium *ΔsopABEE2* mutant which docks to COS7 cells, but fails to trigger membrane ruffling, fails to invade, engages lower numbers of TTSS and requires 2–3 times longer for complete SipA-delivery than wt *S*. Typhimurium [2], [4] (M1252 (pGFPpHilA); *sipA_HA_sptP_M45_ΔsopABEE2*). As expected, this strain required approx. 2-fold longer for completing effector protein injection and the SipA injection was completed well before the SptP injection (t_50%_(SipA) = 215 sec vs. t_50%_(SptP) = 595 sec; p<<0.001, see Materials and Methods; Table 1; suppl. Fig. S2).

Finally, we have verified the hierarchy of injection in wild-type *S*. Typhimurium w/o *hilA* over-expression (M1269 (pGFP); *sipA_M45_sptP_HA_*). Again, the SipA-pool in the bacterial cytosol was depleted sooner than the SptP pool (Table 1). Furthermore, previous work had established that SopE- and SipA-injection occur simultaneously [2]. And additional control experiments with strains carrying epitope-tagged *sopE* and *sptP* verified that SopE was injected earlier than SptP (pHilA; strains *sopE_M45_sptP_HA_* and *sopE_HA_sptP_M45_*; data not shown). Together, these results show that there is a hierarchy of TTSS-1 effector protein injection: SopE and SipA injection are completed soon after host cell contact while SptP injection is completed significantly later.

### Computer simulation of hierarchical effector protein injection via the TTSS-1

The mechanism underlying the transport hierarchy among different TTSS effector proteins was unknown. We have explored plausible mechanisms using a computer simulation approach (described in detail in the Supplemental Material S1).

Earlier work had provided some clues. 1) The number of TTSS-1 effector proteins was much larger than the number of active TTSS-1 systems. Wild type strains (no *hilA* over-expression) harbor approx. 6000 molecules SipA, 3000 molecules SopE, 10000 molecules SptP and only some of the approx. 10-100 TTSS-1 systems are thought to be activated upon host cell encounter (this work, [2], [4], [25]). Similarly, *hilA* over-expressing strains harbor much more effector proteins (including 25000 molecules of SipA, SopE or SptP; this work) than TTSS-1 systems (Sturm, Winnen and Hardt, unpublished). Thus, effector proteins must compete for access to the TTSS. 2) Estimates from virulence-associated and flagellar TTSS suggest that secretion rates are in the range of 10–150 cargo proteins per second [2], [3], [26], [27], [28]. 3) Depletion of the TTSS-1 effector protein pools required between 80–600 sec (Fig. 2C, Tab. 1; [2]). This indicated that competition for the limited number of active TTSS-1 apparatuses might represent the bottleneck determining the over-all transport rate and the hierarchy of injection.

**Figure 3:**
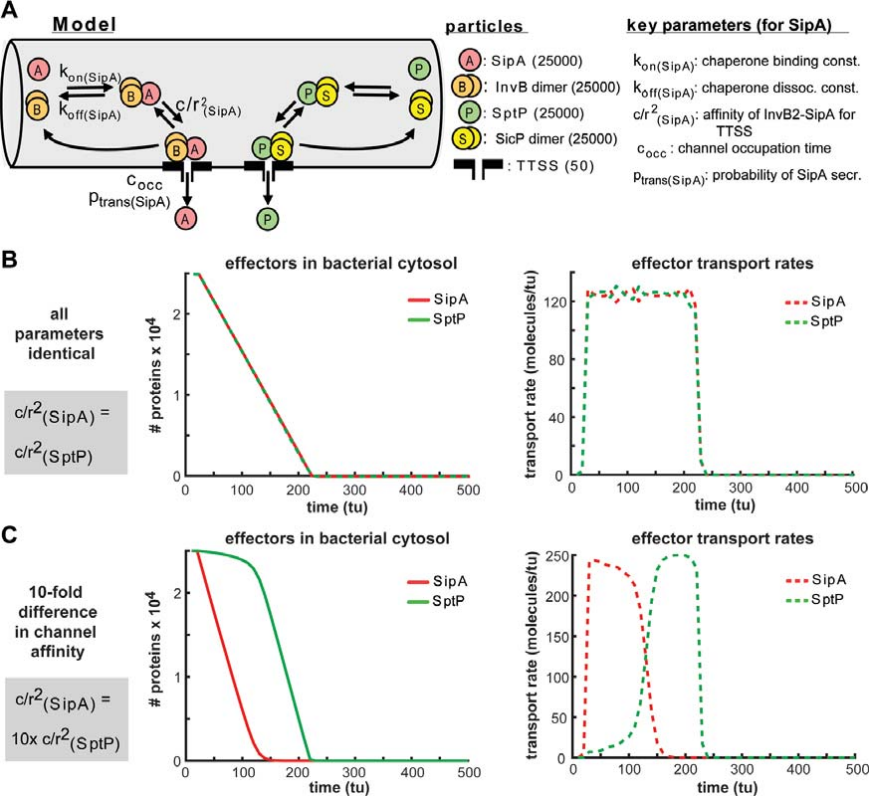
Computer simulation exploring hierarchical SipA and SptP injection by *hilA*-over-expressing bacteria (M1223 or M1269 (pHilA)). (A) Model for TTSS injection of SipA and SptP. Key parameters for SipA binding to the chaperone InvB_2_ and interaction of the InvB_2_-SipA complex to the TTSS are shown. The same types of parameters describe SptP secretion (not shown). Brackets: numbers of particles. (B) Simulation of SipA and SptP secretion assuming identical parameters for both effector proteins. (C) Simulation of SipA and SptP secretion assuming that InvB_2_-SipA has a 10-fold higher affinity for the TTSS than SicP_2_-SptP. All other steps of SipA- and SptP-secretion had identical parameters. In this case, SipA is secreted before SptP. For details of the simulation and additional simulations see Supporting Materials.

We simulated effector protein injection by *hilA* over-expressing bacteria (for bacteria w/o *hilA* over-expression, see Supplemental Material S1). Two different effector proteins (e.g. 25000 SipA and 25000 SptP), their cognate chaperones (e.g. 25000 InvB_2_ and 25000 SicP_2_) and complex formation (of InvB_2_SipA, SicP_2_SptP) were simulated via particles diffusing by “Brownian motion” within a cylindrical space representing the bacterial cytosol. Active TTSS were simulated as a finite number of small “outlets” located in the wall of the cylinder. We assumed that 50 active TTSS were present per *hilA* over-expressing bacterium (Fig. 3A). Formation of a chaperone-effector protein complex was defined as a requirement for productive binding to a TTSS and “injection”. The parameters for forming the effector protein-chaperone complexes, for binding of these complexes to the TTSS, for the probability that binding leads to “injection” and for the time required for “injection” could be adjusted freely (Fig. 3A; see also supplemental online materials). This allowed us to explore a wide range of parameters. As expected, two effector protein-chaperone pairs with identical sets of parameters were “injected” simultaneously by strains with *hilA* over-expression (Fig. 3B; see Suppl. Fig. S3A for data w/o *hilA* over-expression). In contrast, a 10-fold difference in the affinity of the two different chaperone-effector protein complexes for binding to the TTSS, resulted in a hierarchy of transport (c/r^2^_(SipA)_ = 10× c/r^2^_(SptP)_; Fig. 3C; see Suppl. Fig. S3B for data w/o *hilA* over-expression). Thus, the combination of limiting numbers of active TTSS and their different affinities for particular effector protein-chaperone complexes was sufficient to explain an “injection-hierarchy” in type III secretion. This represents a testable working hypothesis and an important topic for future research.

## Discussion

We have studied the early phase of *Salmonella* host cell invasion. Under appropriate environmental conditions, the bacteria express the TTSS-1 apparatus and harbor a pool of pre-formed effector proteins in the cytosol. Earlier work had shown that host cell contact triggers type III secretion and that effector protein injection starts within a few seconds [2]. Here, we found that different effector proteins were injected at different times during this early phase of the bacteria-host interaction. SipA and SopE were injected coequally right after host cell contact. In contrast, SptP injection occurred significantly later.

**Figure 4:**
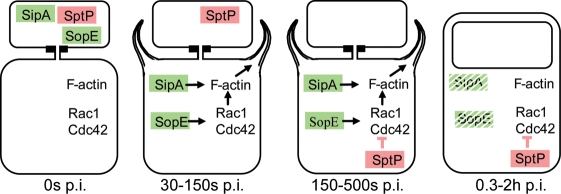
Model for the timing of host cell manipulation by TTSS-1. The bacterium harbors a pre-formed effector protein pool and the TTSS is triggered upon docking to the host cell (0s p.i.). SipA and SopE are injected during the first phase (first 30–150s p.i.) and trigger ruffling and invasion. Afterwards, SptP is injected and begins to reverse Rac1 and Cdc42 activation (150–500s p.i.). Finally, SopE (and presumably SipA) are degraded while SptP persists considerably longer in the host cell cytosol. As a result, the host cell actin cytoskeleton returns back to its normal shape.

We speculated that this injection hierarchy might help to circumvent functional interference between different effector proteins and to optimize host cell manipulation. Very early after host cell contact (30–150s p.i.), SopE and SipA were injected (t_50%_≈100 sec; this work, [2]; Fig. 4). SopE is a G-nucleotide exchange factor activating Cdc42 and Rac1, two key regulators of host cell actin polymerization [10], [29]. SopE cooperates with SipA which binds to F-actin, thus promoting actin polymerization, actin filament stabilization and bundling [30], [31], [32], [33]. The fast SipA- and SopE injection kinetics and their functional cooperation explained why actin rearrangements, membrane ruffling and bacterial engulfment can be triggered within<1 min ([34]; Suppl. Fig. S4). SptP translocation occurred later (t_50%_≈300 sec). SptP has a tyrosine phosphatase domain [35] and a GTPase activating domain which inactivates RhoGTPases like Cdc42 and Rac1 [13]. Thereby, SptP reverses the changes inflicted by SopE, helps to limit pronounced membrane ruffling to the first 20–30 min of the infection and allows the host cells to regain their normal architecture within 0.3–2 hours [36]. Thus, the sequential injection of SipA/SopE and SptP may circumvent functional interference between the effector proteins triggering ruffling and invasion (e.g. SopE, SipA, SopE2, SopB) and those effector proteins serving to silence these early responses at later stages of the infection (e.g. SptP). At these later stages (0.5–2h p.i.), *S*. Typhimurium reverses the host cell cytoskeletal rearrangements. Strikingly, functional interference of effectors at these later stages is avoided by a different mechanism, i.e. differential proteasomal degradation. In the host cell cytoplasm, TTSS-1 effector protein SopE (t_1/2_<15 min) is degraded much faster than SptP (t_1/2_≈60 min; [36]). By 0.5–2h p.i., SopE is degraded, the remaining SptP returns the host cell architecture to its normal state and thereby augments the generation of a permissive environment for intracellular growth of *S*. Typhimurium.

The assembly of the outer parts of the TTSS apparatus also requires transport of different cargo proteins at different times. Here, completion of a particular step in TTSS assembly triggers proteolytic processing of the TTSS and thereby changes substrate specificity. In flagellar TTSS, proteolytic processing of FlhB regulates the proper export/assembly of flagellar components [37], [38] and in *Yersinia* spp., proteolytic processing of the FlhB homolog YscU switches the TTSS from exporting TTSS-apparatus components to effector protein injection [39]. It remains to be analyzed whether other substrate switching events during the activation of type III injection after host cell contact are also attributable to this type of mechanism [40], [41]. Nonetheless, proteolytic cleavage seems to guide major changes in TTSS transport specificity, i.e. switching between stages of TTSS assembly.

Hierarchical effector protein injection represents a new mechanism for fine-tuning host cell manipulation during the early phase of infection when the pre-formed effector protein pool is delivered. In the case of the *S.* Typhimurium TTSS-1, the hierarchical injection of SipA/SopE and SptP avoids functional interference between the different TTSS-1 effector proteins. Other TTSS-1 effector proteins are also involved in this early phase of host cell manipulation. This includes the invasion-mediating effector proteins SopE2 and SopB and the TTSS-1 substrates SipC and SipB. The latter *Salmonella* proteins have dual functions in forming an essential part of the TTSS-1 injection machinery (the “translocon”) and in directly manipulating host cell signaling. It will be of great interest to analyze the timing of their delivery into the host cell, how this may enhance functional cooperation between the effector proteins, how this relates to the dynamic responses of the host cell and how this translates into efficient host cell manipulation. Most likely, the precise timing of host cell manipulation by TTSS is of importance for many bacterial pathogens and symbionts. Thus, hierarchical injection of TTSS effectors may be a common principle governing the early phases of these bacteria-host interactions.

## Materials and Methods

### Bacterial Strains and Plasmids

pHilA is an arabinose-inducible *hilA*-expression plasmid with a pBAD/*Myc*-His backbone (pCH112; pBR322 ori; amp^R^; [14]). pGFP is an erythromycin-resistant derivative of pM965 [15] expressing *gfpmut2* under control of the constitutive *rpsM-*promoter (pM1274; pSC101 ori; erm^R^).

Wild type *S*. Typhimurium SL1344 has been described [16]. Δ*invG* (SB161; [17]) is an SL1344 mutant with a disrupted TTSS-1. M1223 (SL1344, *sipA_HA_ sptP_M45_*) was constructed by allelic exchange of *sipA* with a gene cassette encoding *sipA_HA_* and a kanamycin resistance-gene cassette using the method of Datsenko and Wanner [18] and by chromosomal integration of a suicide plasmid (tet^R^) encoding the C-terminal region of *sptP* fused to the M45 epitope (MDRSRDRLPPFETETRIL) [19]. M1269 (SL1344, *sipA_M45_ sptP_HA_*) was constructed by allelic exchange of *sipA* with a gene cassette encoding *sipA_M45_* and a kanamycin resistance-gene cassette using the method of Datsenko and Wanner [18] and by chromosomal integration of a suicide plasmid (tet^R^) encoding the C-terminal region of *sptP* fused to the HA epitope (YPYDVPDYA). M1252 (SL1344, *sipA_HA_ sptP_M45_ ΔsopABEE2*) was constructed by P22-mediated transduction of *sipA_HA_* and *sptP_M45_* from M1223 into M712 (SL1344, *ΔsopABEE2*; [20]).

*Tsr-venus* was retrieved from pVS152 [21] via *Not*I and *Eco*R47III and cloned into the *Not*I and *Msl*I sites of the suicide plasmid pM1300 [2] downstream of *sipA_M45_*. This suicide plasmid was conjugated into *S*. Typhimurium strain ATCC14028 and recombinants were selected. The resulting strain M2001 carried *tsr-venus* at the 3′-end of the chromosomal *sicAsipBCDA* operon.

Bacteria were grown under TTSS-1 inducing conditions (mild aeration; 12h in LB with 0.3M NaCl, diluted 1:20 into fresh medium and sub-cultured for 4h, 37°C) as described previously [20].

### Immunofluoresecence microscopy

For immuno-staining of effector proteins in the bacterial cytosol (Figs. 1A–C, 2C, 2D, Tab. 1), *S*. Typhimurium immobilized on gelatine coated cover slips ( = no host cell contact) or infection assays were fixed (20 min, 22°C, 4% paraformaldehyde, 4% sucrose, PBS), incubated in 20% sucrose (in PBS, 10 min, 22°C), permeabilized with buffer a (5 min, 22°C, 50 mM EDTA, 20 mM Tris/HCl, 1.8 g/l glucose, 0.1% Triton ×100, pH8) washed 3× in buffer b (22°C, 10 mM EDTA, 25 mM Tris/HCl, 1.8 g/l glucose, pH8), and incubated for 1 h in buffer b (supplemented with 5 g/l lysozyme; 4°C) as described [2].

*S*. Typhimurium was stained using DAPI and/or a polyclonal rabbit α-*Salmonella* O-1,4,5,12(8) antiserum (Difco) and polyclonal goat α-rabbit-Cy5 conjugate (Fig. 2). SipA_M45_, SipA_HA_, SptP_M45_ and SptP_HA_ were stained using monoclonal mouse α-M45 (kindly provided by P. Hearing) or polyclonal rabbit α-HA (Santa Cruz) and polyclonal goat α-mouse-Cy3 (Fig. 1A) or –rhodamine (Fig. 1C) and polyclonal goat α-rabbit-FITC (Fig. 1A) or –Cy5 (Fig. 1C) conjugates (Jackson Immuno Research). Images were taken with a confocal system (PerkinElmer/Zeiss; see below).

### FACS analysis

The *sipA* expression level of *S*. Typhimurium strains carrying the *tsr*-*venus* fusion (e.g. M2001; Fig. 1D) was determined by FACS analysis. The bacteria were grown under TTSS-1 inducing conditions (see, above). Afterwards, the cultures were supplemented with streptomycin (50 µg/ml) for 2 hours to prevent de novo protein biosynthesis while allowing complete maturation of the fluorescent reporter protein. FACS analysis was performed using a FACSCalibur (Becton Dickinson) equipped with a 488 nm laser. The *tsr*-*venus* emission was analyzed at 530 nm. Bacteria were identified by side scatter (SSC).

### Quantitative Western blot analysis of the effector protein load per bacterium

Bacteria were grown under TTSS-1 inducing conditions. M45- or HA-tagged SipA, SopE or SptP present in the bacterial cell pellet was detected by Western blotting using a monoclonal mouse-α-M45-, or a polyclonal rabbit-α-HA antibody and the signal was calibrated by comparison with known amounts of purified SipA_M45_ or SipA_HA_ fusion proteins present on the same blot (see supp. Fig. S1). The number of bacteria loaded per lane was determined by plating on LB agar. Samples of the culture were immobilized on gelatine-coated coverslips and the fraction of *sipA-* or *sptP-* expressing bacteria was analyzed as shown in Fig. 1A and C.

The number of effector proteins per expressing bacterium was determined in at least three independent assays and calculated by dividing the average number of effector molecules per bacterium (result from quantitative Western blot) by the fraction of effector-expressing bacteria (results from cover-slips; [2]).

### Real time imaging

To assay depletion of intra-bacterial effector proteins, time lapse microscopy was performed as described, recently [2]. Briefly, COS7 cells were seeded onto a glass-bottom culture dish in DMEM (PAA Laboratories; 5% FCS) and grown to 70–80% confluency. Cells were mounted on a temperature controlled stage (37°C) and infected with *S.* Typhimurium in the presence of 30 µg/ml chloramphenicol. The infection (MOI = 25) was imaged in phase contrast and GFP fluorescence channels (10 frames/min; 40× objective). After 10, 15 or 20 min of infection the cells were fixed, permeabilized, treated with lysozyme, immuno-stained for SipA, SptP and LPS (Fig. 2) and imaged by confocal microscopy (see above).

For each bacterium, we determined the status of effector-depletion from the bacterial cytosol (“full” = ongoing/little/no injection vs. “empty” = advanced/completed injection, see Fig. 2A,C) and the time between host cell docking and fixation ( = “injection time”, see Fig. 2B). Effector depletion data were analyzed by a “rolling average” algorithm (see Fig. 2D): For each infection time point, we determined the fraction: (full bacteria)/(all bacteria) at this time point ±75s ( = 150s interval centered around the particular injection time). The rolling average was analyzed separately for SipA and SptP. This yielded the t_50%_ values ( = 50% of the bacteria had completed injection of a particular effector; see Tab. 1). Data between the t_50%_(SipA) and t_50%_(SptP) time points were analyzed statistically: values of 0 and 1 were assigned to “full” (ongoing/little/no injection = 1) vs. “empty” (advanced/completed injection = 0) bacteria, and the values for SipA- and SptP-injection by a particular strain were compared using the Mann-Whitney U test.

We have estimated the threshold of detecting effector proteins in the bacterial cytosol. An upper estimate for this can be obtained from SopE_M45_ levels in bacteria w/o *hilA* over-expression. These bacteria harbor 1000–3000 molecules of SopE_M45_ in the cytosol ([2]; this work). This number of effector proteins could be detected very reliably inside the bacterial cytosol. The detection was reliable for SopE, SipA and SptP (ranges from 1000 to 25000 molecules per bacterium). Now, let's consider a pHilA expressing strain loaded with approx. 25000 molecules of a particular effector protein before host cell encounter. We estimate that the assay system would reliably detect the bacterium as “loaded” if injection is <90% completed.

### Computer simulation of effector protein secretion

The effectors SipA and SptP and their cognate chaperone dimers InvB_2_ and SicP_2_ were simulated as particles diffusing by Brownian motion in the bacterial cytosol. Interactions between effectors and chaperones and binding of chaperone-effector complexes to the TTSS-1 was simulated according to fixed parameters as described in detail in the Supporting Material. The bacterial cytosol was represented as a cylindrical space and the TTSS-1 as “outlets” on the wall of this cylinder. Particle diffusion, interactions and secretion (via TTSS-1) from the bacterium was simulated in short time intervals, and the number of SipA- and SptP-molecules remaining in the bacterial cytosol and the rate of SipA- and SptP-secretion were plotted as a function of time (Fig. 3B and C; see Supporting Material for details).

## Supporting Information

Supplementary Material S1 Supporting online material: describes the computer model for TTSS effector injection also contains additional experimental data
(0.17 MB DOC)

Figure S1 A. Host cell invasion by hilA over-expressing S. Typhimurium strains. COS7 tissue culture cells were infected (moi = 10) for 50 min with the indicated strains and the invasiveness was analyzed in a gentamycin protection assay, as described [3]. The invasiveness was normalized with respect to the number of wild type S. Typhimurium recovered from within the Cos7 cells. A mutant with a disrupted TTSS-1 apparatus (SB161, ΔinvG; [4]) served as a negative control. The data were derived from three independent experiments. They verified that hilA over-expression did not impair TTSS-1 function. B. Typical quantitative Western blot for analyzing the number of effector proteins present per TTSS-1 expressing bacterium. The intensities were scanned and analyzed as described in Materials and Methods. The numbers below the blot indicate the numbers of bacteria of the culture (colony forming units; grown under TTSS-1 inducing conditions) which were loaded onto the respective lane. Data from at least three experiments like this were averaged for each strain and each bacterial protein, analyzed.
(1.83 MB TIF)

Figure S2 Time course of SipA- and SptP-injection by S. Typhimurium M1252(pHilA). M1252 is an isogenic derivative of M1223 which lacks the key, invasion-mediating effector proteins (sipA_M45_sptP_HA_ ΔsopABEE2). COS7 cells were infected with M1252(pHilA) and the infection was monitored by time lapse phase contrast microscopy as described in Fig. 2. Cells were fixed, permeabilized with lysozyme, and immuno-stained for LPS (blue), intra-bacterial SipA (red) and intra-bacterial SptP (green). For each bacterium, the graph shows the time between docking and fixation as well as the presence/absence of SipA (red) and SptP (green) in the bacterial cytosol. Gray lines connect SipA and SptP data from the same bacterium. The data was fitted using a rolling average algorithm (red and green lines, see Materials and Methods) to determine when injection was completed with 50% probability (t50%).
(4.42 MB TIF)

Figure S3 Computer simulation exploring hierarchical SipA and SptP injection by wild type bacteria (e.g. M1269; no hilA over-expression). (A) Simulation of SipA and SptP secretion assuming identical parameters for both effector proteins. Please note that the average “active” wild type S. Typhimurium harbors approx. 10000 molecules of SptP and 6000 molecules of SipA in the cytosol. (B) Simulation of SipA and SptP secretion assuming that SipA-InvB2 has a 10-fold higher affinity (c/r2) for the TTSS than SptP-SicP2. All other steps of SipA- and SptP-secretion had identical parameters. In this case, the bulk of SipA is secreted before SptP.
(5.77 MB TIF)

Figure S4 Time course of TTSS-1 induced membrane ruffling. MDCK tissue culture cells were infected with wild type S. Typhimurium. The infection process was monitored on a temperature-controlled stage by phase contrast time lapse microscopy. These data illustrate that membrane ruffling is induced within the first 30–60 seconds after the bacterium has docked to the host cell. The outline of the cell is indicated by the dashed line.
(1.59 MB TIF)
