# Supplementary material for: Hierarchical Effector Protein Transport by the Salmonella Typhimurium SPI-1 Type III Secretion System
Source: PLoS One. 2008 May 14;3(5):e2178. doi: 10.1371/journal.pone.0002178 (PMC2364654; doi:10.1371/journal.pone.0002178)
Supplement: Supplementary Material S1 — Supporting online material: describes the computer model for TTSS effector injection also contains additional experimental data (0.17 MB DOC) [file pone.0002178.s001.doc]

**Supplementary Material S1**

**Table of contents:**

**A) Computer modeling of SipA and SptP injection** ……………………..... 2

A.1. Simulating TTSS-1 effector protein injection …………………… 2

A.2. Computer model to simulate TTSS-1 effector protein injection … 2

A.3. Results obtained by the computer simulation …………………… 5

**B) Supplementary Tables S1, S2, S3** ……………………………………….. 8

**C) Supplementary Figure legends** ………………………………………….. 11

**D) Supplementary References**……………………………………………….. 12

**A) Computer modeling of SipA and SptP injection**

**A.1. Simulating TTSS-1 effector protein injection**

To explore mechanisms underlying a transport hierarchy among different TTSS-1 effectors, we have used a computational model to simulate the TTSS-1 injection of two different effectors. The model simulates the formation/dissociation of chaperone-effector complexes, binding of these complexes to the transport channels and the transport of effectors out of the bacterial cytosol (=”injection”).

Based on our results (Fig. 2), SipA and SptP together with their cognate chaperones (InvB and SicP, respectively) were chosen as candidate effectors for the computer simulation. Biochemical quantification of the numbers of SipA, SptP and InvB molecules per bacterium and our data on SipA-InvB complex formation and dissociation ([1] and this work) served as a basis for estimating reasonable simulation parameters for effector protein secretion by TTSS-1. These parameters differ somewhat between bacteria over-expressing *hilA* and bacteria that do not. The former situation (*hilA* over-expression) has been explored in the simulations presented in Fig. 3. And the latter situation has been studied in the simulations presented in the Supplementary Fig. S3. Notably, in both situations, a 10-fold difference in the channel affinity (c/r^2^) of SipA-InvB_2_ and SptP-SicP_2_ was sufficient to yield hierarchical injection of SipA and SptP (Fig. 3; Supplementary Fig. S3).

**A.2. Computer model to simulate TTSS-1 effector protein injection**

The model for TTSS-1 effector protein injection was implemented by simulating two different effector proteins and their cognate chaperone dimers. For simplicity, chaperone dimer formation is not considered in the model. In vitro, the chaperones InvB and SicP are thought to form stable dimers. Therefore, the chaperone dimers are simulated as single particles for the purpose of this simulation. Unbound effector proteins, chaperone dimers and effector protein-(chaperone)_2_ complexes were simulated as particles diffusing by Brownian motion within a cylindrical space representing the bacterial cytosol.

For this purpose, six types of particles were simulated to move within the cylindrical space according to:

$x_{i}(t+\Delta t)=x_{i}(t)+\sqrt{\Delta t*2*diff}*R_{n}$, (1)

where *diff* represents the diffusion constant of the particle and *R_n_* is a random, standard Gaussian distributed number.

The six types of particles represent:

1. free effector protein 1 (SipA free)

2. free chaperone dimer 1 (InvB_2_ free)

3. effector-chaperone complex 1 (SipA-InvB_2_)

4. free effector protein 2 (SptP free)

5. free chaperone dimer 2 (SicP_2_ free)

6. effector-chaperone complex 2 (SptP-SicP_2_)

***Simulation of effector-chaperone complex formation and dissociation:***

An effector molecule binds to its cognate chaperone if the distance between the two particles is smaller than the specific binding distance for the respective chaperone-effector pair (parameters *bindSipAInvB_2_* or *bindSptPSicP_2_*; simulate the binding constants k_on(SipA)_ and k_on(SptP)_). If more than one interaction partner is available at a given time, the one with the smallest distance from its binding partner is considered for complex formation.

Chaperone-effector complexes dissociate within a time interval with a probability of

$k_{off}=1-\exp\left(-\frac{\Delta t}{sep}\right)$, (2)

where *sep* represents the average lifetime of the complex (model parameters *sepSipAInvB_2_* or *sepSptPSicP_2_*).

Thus, the rates of complex formation and dissociation can be adjusted by choosing proper values for the model parameters *bind* and *sep* for the respective effector-chaperone pair. In biochemistry, the closely related rate constants k_on_ and k_off_ are generally used to describe complex formation. As most readers will be familiar with these constants, we have used the rate constants k_on_ and k_off_ in Fig. 3A.

***Simulation of effector protein injection via the TTSS-1*:**

Effector protein injection occurs if a chaperone-effector complex binds productively to a TTSS-1 apparatus (channel). In the simulation, the affinity of the chaperone-effector complex for the channel is represented as follows: Complexes can interact with a channel once their distance from a channel site is smaller than the distance parameter *r*. Of all complexes that fulfill this condition, the complex for which the term *c*/*r*^2^ is maximal will interact with the channel. c represents the probability of complex formation. The parameters c and r are complex-specific and c/r^2^ represents the affinity of a particular effector-chaperone complex for TTSS-1. Therefore, c/r^2^ allows simulating the competition for the limiting number of induced TTSS-1.

Due to the interaction of a complex with a channel, this channel becomes occupied for a time interval determined by the parameter *c_occ_*. During this time, the channel is unavailable for further interactions.

Upon interaction of a complex with a channel, the effector protein is transported out of the bacterial cytosol (and injected into the host cell) with a probability of *p_trans_*. Thus, if productive transport occurs, the chaperone-effector complex dissociates and yields a free chaperone, whereas due to transport the effector particle is no longer present in the bacterial cytosol. The transport probability *p_trans_* may be considered as inversely proportional to the “activation energy” for the channel (and the associated ATPase) to dissociate the effector-chaperone complex and initiate the injection process. If no transport occurs (i.e. when *p_trans_* < 1), the effector-chaperone complex dissociates from the TTSS.

***Timing and parameters for the model simulation:***

The processes of particle movement (see equation 1), complex formation/dissociation (see equation 2) and interactions with TTSS-1 apparatuses (channels) are simulated in discrete time intervals Δ*t*, which are chosen such that all processes are temporally resolved. The relevant parameters for the computer simulation are listed in Supplementary Tab. S1.

***Initial simulation settings:***

Here we describe the parameter settings for simulating effector protein injection by wild type *S*. Typhimurium. Differences between wild type and *hilA* over-expressing bacteria are mentioned in the appropriate paragraphs.

1) Diffusion constants

Small differences in the diffusion constants between the two simulated effectors did not significantly affect their transport hierarchy. Thus, as a simple approximation the diffusion constants for all particles were set to be identical for the purpose of this simulation.

2) Binding and separation constants for the SipA-InvB_2_ complex

In a previous study, we had estimated the association and dissociation rates for the formation of SipA-InvB_2_ complexes by surface plasmon resonance experiments (Schlumberger *et al*., 2005). These data served as a basis to determine the number of SipA-InvB complexes formed in the bacterial cytosol under equilibrium conditions (before initiation of transport). The simulation parameters *bindSipAInvB_2_* and *sepSipAInvB_2_* have been set accordingly to match the number of complexes and free SipA and InvB_2_ particles found under equilibrium conditions. In the simulation, equilibrium conditions were achieved by leaving the transport channels closed for a time interval of *eqtime*, which was sufficient for the processes of complex formation to reach equilibrium state.

3) Number of particles

The number of effector protein molecules used for the simulation was directly derived from our data from the quantitative Western blots. In wild-type *Salmonella* strains (no *hilA* over-expression), only ~15-25% of the bacterial population expresses the effectors SipA and SptP at detectable levels. Our simulation considered such an “active” member of the *S.* Typhimurium population. The bacterium harbored ~6000 molecules SipA and ~10000 molecules SptP (this work and Schlumberger *et al*., 2005). We have also estimated the number of InvB dimers in wild-type bacteria (~6000 dimers/bacterium; K. Ehrbar, I. Duss and W.-D. Hardt, unpublished data). As an approximation, the number of SicP_2_ molecules was estimated to be in the range of the numbers of SptP molecules (i.e. 10000; Supplementary Table S2).

For the *S.* Typhimurium strains over-expressing the key TTSS-1 regulator HilA (data presented in Fig. 3), the particle numbers used for the simulation was adjusted according to the values determined by quantitative Western blotting, i.e. 25000 molecules SipA, 25000 molecules SptP, 25000 InvB_2_ particles and 25000 SicP_2_ particles.

4) Number of channels

The number of channels used for the simulation does only affect overall transport capacity of a bacterium but has no influence on the relative transport rates of the individual effectors. This parameter (*nbrC*) was set to the value of 20 channels per bacterium for all simulations of wild type bacteria (no *hilA* over-expression). This is in the range that has been estimated previously by Kubori *et al*. [2] and our work [1]. Kubori *et al.* found by electron microscopy imaging, that an average *S*. Typhimurium cell harbored 10-100 TTSS “needle complexes” per cell. And our earlier work had established that SipA is delivered into the host cell as 3-15 SipA foci located right at the bacteria-host cell interface. This suggested that the SipA arriving in a particular SipA focus might be transported through one channel, or a set of a few adjacent channels.

For *hilA* over-expressing strains, *nbrC* was set to 50 channels per bacterium. This estimate was based on the observation, that *hilA* over-expressing bacteria express TTSS-1 apparatus genes at approx. 2-3 fold increased levels (Sturm and Hardt, unpublished observations). It should be noted that this is a rough estimate. More work will be required to generate quantitative information. However, provided that the number of TTSS-1 channels remains limiting, the key observation (transport hierarchy between SopE/SipA and SptP) is not affected by small changes in the numbers of channels available for transport.

5) Channel occupation time

In an actively transporting bacterium (right after docking to the host cell), virtually all channels are expected to be occupied due to transport activity, as effector-chaperone complexes vastly outnumber the active channels of the bacterium. Only under these conditions, the different effector-chaperone complexes will compete for binding to available channels, and therefore a hierarchical effector protein transport can be established.

Furthermore, the channel occupation time (*c_occ_*) determines the overall transport capacity per bacterium. Thus, under the condition that free channel availability is limiting, *c_occ_* is inversely proportional to the transport rates for the effector proteins. *c_occ_* was set to the same value for SipA- and SptP injection.

6) Effector-specific channel parameters

In general, the effector-specific parameters (binding constants, binding distances and transport probabilities for the chaperone-effector complexes) are unknown. Therefore, a key objective of this simulation was to explore if effector-specific differences in these parameters can explain a transport hierarchy as observed in our experiments.

The affinity of effector-chaperone complexes to a transport channel is defined in the model by the factor *c*/*r*^2^. For simplicity, we have set the channel binding distance r (*rSipA* or *rSptP*) identical for the two different effector-chaperone complexes to be analyzed. The value for r was identical in all simulations, presented. Differences in affinity between the different complexes and transport channels were simulated by adjusting the channel binding constants (*cSipA*, *cSptP*). Finally, we initially assumed that all interactions between effector-chaperone complexes and transport channels lead to transport of the effector (*p_trans(SipA)_* = *p_trans(SptP)_* = 1).

In pilot simulation studies, we have adjusted *c_occ_* and *cSipA* such that the obtained “virtual” SipA transport rates match our experimental data for SipA transport (see [1] and this work).

**A.3. Results obtained by the computer simulation.**

*Parameter conditions tested using the model simulation:*

Generally, we explored three different conditions for the effector-specific parameters with our model simulation:

1. All effector-specific parameters identical for the two different effectors
2. Different channel binding constants (*cSipA* > *cSptP*)
3. Different transport probability (*p_trans(SipA)_* > *p_trans(SptP)_*)

For conditions 2 and 3, we tested the effects of a 10-fold (a) and a 4-fold (b) difference in the respective parameters for SipA and SptP on the transport hierarchy.

Before the TTSS channels were opened in the simulation, effector-chaperone complex formation quickly reached an equilibrium state where the majority of effector molecules are bound to their cognate chaperone dimers. This equilibrium state reflects the stability of effector-chaperone complexes measured *in vitro* [1]. After channel opening, effector transport started. Thereby, the effectors are gradually depleted from the bacterial cytoplasm and free chaperone dimers are generated. The low levels of free effector molecules remained fairly constant during transport under all conditions, analyzed (not shown).

**A.3.a. Results for *hilA* over-expressing strains:**

In the main body of the paper, we have presented the results obtained simulating *hilA* over-expressing strains (Fig. 3B, C; for parameters see Supplementary Tab. S2 and S3).

**A.3.b. Results for wild type *S*. Typhimurium (no *hilA* over-expression)**

In the supplement we present the results obtained by simulating wild type *S*. Typhimurium (no *hilA* over-expression). The initial parameters used for these simulations are listed in Supplementary Tab. S2.

*A.3.b.1. Identical parameters for both effector-chaperone pairs*

As expected, if all simulation parameters had identical values for the different effector-chaperone pairs, the two effectors were “injected” at the same time. In this case, the observed transport rates were proportional to the total number of effector protein/chaperone molecules. Due to the limiting number of transport-competent channels, both effectors were co-transported at constant rates until the pre-formed effector pools in the bacterial cytoplasm were depleted (Supplementary Fig. S3A).

*A.3.b.2. Different channel binding constants (c/r^2^_(SipA)_ > c/r^2^_(SptP)_)*

A 10-fold difference in the affinity of the effector-chaperone complexes for the TTSS resulted in hierarchical transport (Supplementary Fig. S3B). During the first phase of transport, complexes with lower affinity (SptP-SicP_2_) were unable to compete with the SipA-InvB_2_ complex for binding to transport-competent channels. Thus, the complexes with lower affinity for the TTSS were only efficiently transported after the bulk of SipA was depleted from the bacterial cytosol (see Supplementary Fig. S3B). Similar results were obtained using merely a 4-fold difference in channel affinity for the different effector-chaperone pairs (data not shown).

*A.3.b.3. Different transport probabilities (p_trans(SipA)_ > p_trans(SptP)_)*

Next we analyzed the effect of different transport probabilities for SipA and SptP. We set the transport probability *p_trans(SipA)_* at 10x the value of *p_trans(SptP)_*. However, both effector-chaperone complexes (SipA-InvB_2_ and SptP-SicP_2_) had the same channel affinity (c/r^2^; Supplementary Tab. S3). In this situation, the transport of SipA and SptP started at the same time. However, the transport rates were proportional to the transport probabilities. Thus, SptP was depleted from the bacterial cytosol much slower than the competing effector SipA, resulting in hierarchical transport (data not shown). Again, similar results were obtained when we repeated this simulation with *p_trans(SipA)_* = 4x *p_trans(SptP)_*.

*Conclusion:*

Computer modeling revealed that two assumptions were sufficient to explain the injection hierarchy of SipA and SptP: 1) a large number of SipA and SptP molecules compete for transport via a limiting number of TTSS; and 2) the TTSS recognize SipA-InvB_2_ more efficiently than SptP-SicP_2_.

It should be noted that the available experimental data does not allow to determine which aspect of the effector-TTSS interaction (*c/r^2^* or *p_trans_* or both) drives hierarchical transport. Future work will have to address this issue. Nonetheless, both mechanisms imply that SipA-InvB_2_ interacts with the TTSS more efficiently than SptP-SicP_2_. Thus, in the present manuscript we have simply stated that the “interaction efficiency” between SipA and the TTSS differs from that of SptP. This represents a novel concept allowing the precise timing of host cell manipulation by TTSS.

**B) Supplementary Tables S1, S2, S3**

**Supplementary Table S1: Simulation parameter descriptions**

| ***General parameters*** | |
| --- | --- |
| l(x,y,z) | size of the cylindrical shape (x=length; y,z=diameters); (see Fig. 3A) |
| *t* | simulation time |
| Δ*t* | time interval |
| Logint | logging interval (frequency of data output) |
| *eqtime* | simulation time used to reach equilibrium of complex formation (during this time channels are closed) |
| ***Particles in bacterial cytosol*** | |
| *nbrSipA* | number of free SipA particles |
| *nbrInvB_2_* | number of free InvB_2_ particles |
| *nbrSipAInvB_2_* | number of SipA-InvB_2_ complexes |
| *nbrSptP* | number of free SptP particles |
| *nbrSicP_2_* | number of free SicP_2_ particles |
| *nbrSptPSicP_2_* | number of SptP-SicP_2_ complexes |
| ***Diffusion constants for the individual particles in the bacterial cytosol*** | |
| *diffSipA* |  |
| *diffInvB_2_* |  |
| *diffSipAInvB_2_* |  |
| *diffSptP* |  |
| *diffSicP_2_* |  |
| *diffSptPSicP_2_* |  |
| ***Chaperone-Effector complex formation and dissociation*** | |
| *bindSipAInvB_2_* | binding distance for SipA-InvB_2_ complex formation (🡪 *k*_on(SipA)_) |
| *bindSptPSicP_2_* | binding distance for SptP-SicP_2_ complex formation (🡪 *k*_on(SptP)_) |
| *sepSipAInvB_2_* | average lifetime of a SipA-InvB_2_ complex (🡪 *k*_off._) |
| *sepSptPSicP_2_* | average lifetime of a SptP-SicP_2_ complex (🡪 *k*_off._) |
| ***Channel parameters*** | |
| *nbrC* | number of active channels (TTSS-1 apparatuses per bacterium) |
| *cSipA* | probability of SipA-InvB_2_ complex binding to a channel |
| *cSptP* | probability of SptP-SicP_2_ complex binding to a channel |
| *rSipA* | distance parameter for binding of SipA-InvB_2_ to a channel |
| *rSptP* | distance parameter for binding of SptP-SicP_2_ to a channel |
| *p_trans(SipA)_* | probability of transport for SipA |
| *p_trans(SptP)_* | probability of transport for SptP |
| *c_occ_* | channel occupation time upon binding of a complex |

The parameters listed in Supplementary Tab. S1 are considered as relative values with respect to the reference parameters *diffSipA* and *bindSipAInvB_2_* and therefore have dimensionless units.

**Supplementary Table S2: Initial parameter settings for wt *S*. Typhimurium*__**

| ***Description*** | ***parameter*** | ***value*** |
| --- | --- | --- |
| - domain size (x,y,z) | *l* | 2,1,1 |
| - simulation time | *t* | 500 |
| - time interval | Δ*t* | 0.01 |
| - loginterval | *logint* | 10 |
| - simulation time before channel opening used to reach equilibrium state | *eqtime* | 20 |
| - number of particles | *nbrSipA* | 6000 |
|  | *nbrInvB_2_* | 6000 |
|  | *nbrSipAInvB_2_* | 0 |
|  | *nbrSptP* | 10000 |
|  | *nbrSicP_2_* | 10000 |
|  | *nbrSptPSicP_2_* | 0 |
| - number of channels | *nbrC* | 20 |
| - channel occupation time | *c_occ_* | 0.2 |
| - diffusion constants | *diffSipA* | 1 |
|  | *diffInvB_2_* | 1 |
|  | *diffSipAInvB_2_* | 1 |
|  | *diffSptP* | 1 |
|  | *diffSicP_2_* | 1 |
|  | *diffSptPSicP_2_* | 1 |
| - complex binding distances (k_on_) | *bindSipAInvB_2_* | 0.1 |
|  | *bindSptPSicP_2_* | 0.1 |
| - separation time coefficients (k_off_) | *sepSipAInvB_2_* | 1 |
|  | *sepSptPSicP_2_* | 1 |
| - channel binding constants | *cSipA* | 1 |
|  | *cSptP* | 1 |
| - channel binding distances | *rSipA* | 0.1 |
|  | *rSptP* | 0.1 |
| - transport probabilities | *p_trans(SipA)_* | 1 |
|  | *p_trans(SptP)_* | 1 |

***** no *hilA* over-expression; *c/r^2^_(SipA)_* = *c/r^2^_(SptP)_*; *p_trans(SipA)_* = *p_trans(SptP)_*

**Supplementary Table S3: Parameter settings for the different test conditions**

| ***Test condition*** | ***Description*** | ***parameter*** | ***value*** |
| --- | --- | --- | --- |
| *Wild-type S. Typhimurium* | |  |  |
|  | - Particle numbers | *nbrSipA* | 6000 |
|  |  | *nbrInvB_2_* | 6000 |
|  |  | *nbrSipAInvB_2_* | 0 |
|  |  | *nbrSptP* | 10000 |
|  |  | *nbrSicP_2_* | 10000 |
|  |  | *nbrSptPSicP_2_* | 0 |
|  | - Number of channels | *nbrC* | 20 |
| *hilA-overexpressing S. Typhimurium* | |  |  |
|  | - Particle numbers | *nbrSipA* | 25000 |
|  |  | *nbrInvB_2_* | 25000 |
|  |  | *nbrSipAInvB_2_* | 0 |
|  |  | *nbrSptP* | 25000 |
|  |  | *nbrSicP_2_* | 25000 |
|  |  | *nbrSptPSicP_2_* | 0 |
|  | - Number of channels | *nbrC* | 50 |
|  |  |  |  |
| *1) Identical parameters for both effector-chaperone pairs* | | | |
|  | - complex binding distances | *bindSipAInvB_2_* | 0.1 |
|  |  | *bindSptPSicP_2_* | 0.1 |
|  | - channel binding constants | *cSipA* | 1 |
|  |  | *cSptP* | 1 |
|  | - channel binding distances | *rSipA* | 0.1 |
|  |  | *rSptP* | 0.1 |
|  | - transport probabilities | *p_trans(SipA)_* | 1 |
|  |  | *p_trans(SptP)_* | 1 |
| *2) Different channel binding constants* | | | |
| a) | - channel binding constants | *cSipA* | 1 |
|  |  | *cSptP* | 0.1 |
| b) | - channel binding constants | *cSipA* | 1 |
|  |  | *cSptP* | 0.25 |
| *3) Different transport probabilities (data not shown)* | | | |
| a) | - transport probabilities | *p_trans(SipA)_* | 1 |
|  |  | *p_trans(SptP)_* | 0.1 |
| b) | - transport probabilities | *p_trans(SipA)_* | 1 |
|  |  | *p_trans(SptP)_* | 0.25 |

**C) Supplementary Figure legends**

**Supplementary Fig. S1.** A. Host cell invasion by *hilA* over-expressing *S*. Typhimurium strains. COS7 tissue culture cells were infected (moi = 10) for 50 min with the indicated strains and the invasiveness was analyzed in a gentamycin protection assay, as described [3]. The invasiveness was normalized with respect to the number of wild type *S*. Typhimurium recovered from within the Cos7 cells. A mutant with a disrupted TTSS-1 apparatus (SB161, *ΔinvG*; [4]) served as a negative control. The data were derived from three independent experiments. They verified that *hilA* over-expression did not impair TTSS-1 function.

B. Typical quantitative Western blot for analyzing the number of effector proteins present per TTSS-1 expressing bacterium. The intensities were scanned and analyzed as described in Materials and Methods. The numbers below the blot indicate the numbers of bacteria of the culture (colony forming units; grown under TTSS-1 inducing conditions) which were loaded onto the respective lane. Data from at least three experiments like this were averaged for each strain and each bacterial protein, analyzed.

**Supplementary Fig. S2.** Time course of SipA- and SptP-injection by *S*. Typhimurium M1252(pHilA). M1252 is an isogenic derivative of M1223 which lacks the key, invasion-mediating effector proteins (*sipA_M45_sptP_HA_* *ΔsopABEE2*). COS7 cells were infected with M1252(pHilA) and the infection was monitored by time lapse phase contrast microscopy as described in Fig. 2. Cells were fixed, permeabilized with lysozyme, and immuno-stained for LPS (blue), intra-bacterial SipA (red) and intra-bacterial SptP (green). For each bacterium, the graph shows the time between docking and fixation as well as the presence/absence of SipA (red) and SptP (green) in the bacterial cytosol. Gray lines connect SipA and SptP data from the same bacterium. The data was fitted using a rolling average algorithm (red and green lines, see Materials and Methods) to determine when injection was completed with 50% probability (t_50%_).

**Supplementary Fig. S3.** Computer simulation exploring hierarchical SipA and SptP injection by wild type bacteria (e.g. M1269; no *hilA* over-expression). (A) Simulation of SipA and SptP secretion assuming identical parameters for both effector proteins. Please note that the average “active” wild type *S.* Typhimurium harbors approx. 10000 molecules of SptP and 6000 molecules of SipA in the cytosol. (B) Simulation of SipA and SptP secretion assuming that SipA-InvB_2_ has a 10-fold higher affinity (c/r^2^) for the TTSS than SptP-SicP_2_. All other steps of SipA- and SptP-secretion had identical parameters. In this case, the bulk of SipA is secreted before SptP.

**Supplementary Fig. S4.** Time course of TTSS-1 induced membrane ruffling. MDCK tissue culture cells were infected with wild type *S.* Typhimurium. The infection process was monitored on a temperature-controlled stage by phase contrast time lapse microscopy. These data illustrate that membrane ruffling is induced within the first 30-60 seconds after the bacterium has docked to the host cell. The outline of the cell is indicated by the dashed line.

**D) Supplementary References**

1. Schlumberger MC, Muller AJ, Ehrbar K, Winnen B, Duss I, et al. (2005) Real-time imaging of type III secretion: Salmonella SipA injection into host cells. Proc Natl Acad Sci U S A 102: 12548-12553.

2. Kubori T, Matsushima Y, Nakamura D, Uralil J, Lara-Tejero M, et al. (1998) Supramolecular structure of the *Salmonella typhimurium* type III protein secretion system. Science 280: 602-605.

3. Ehrbar K, Winnen B, Hardt WD (2006) The chaperone binding domain of SopE inhibits transport via flagellar and SPI-1 TTSS in the absence of InvB. Mol Microbiol 59: 248-264.

4. Kaniga K, Bossio JC, Galan JE (1994) The Salmonella typhimurium invasion genes invF and invG encode homologues of the AraC and PulD family of proteins. Mol Microbiol 13: 555-568.
