# Supplementary figures and images for: Hierarchical Effector Protein Transport by the Salmonella Typhimurium SPI-1 Type III Secretion System
Source: PLoS One. 2008 May 14;3(5):e2178. doi: 10.1371/journal.pone.0002178 (PMC2364654; doi:10.1371/journal.pone.0002178)

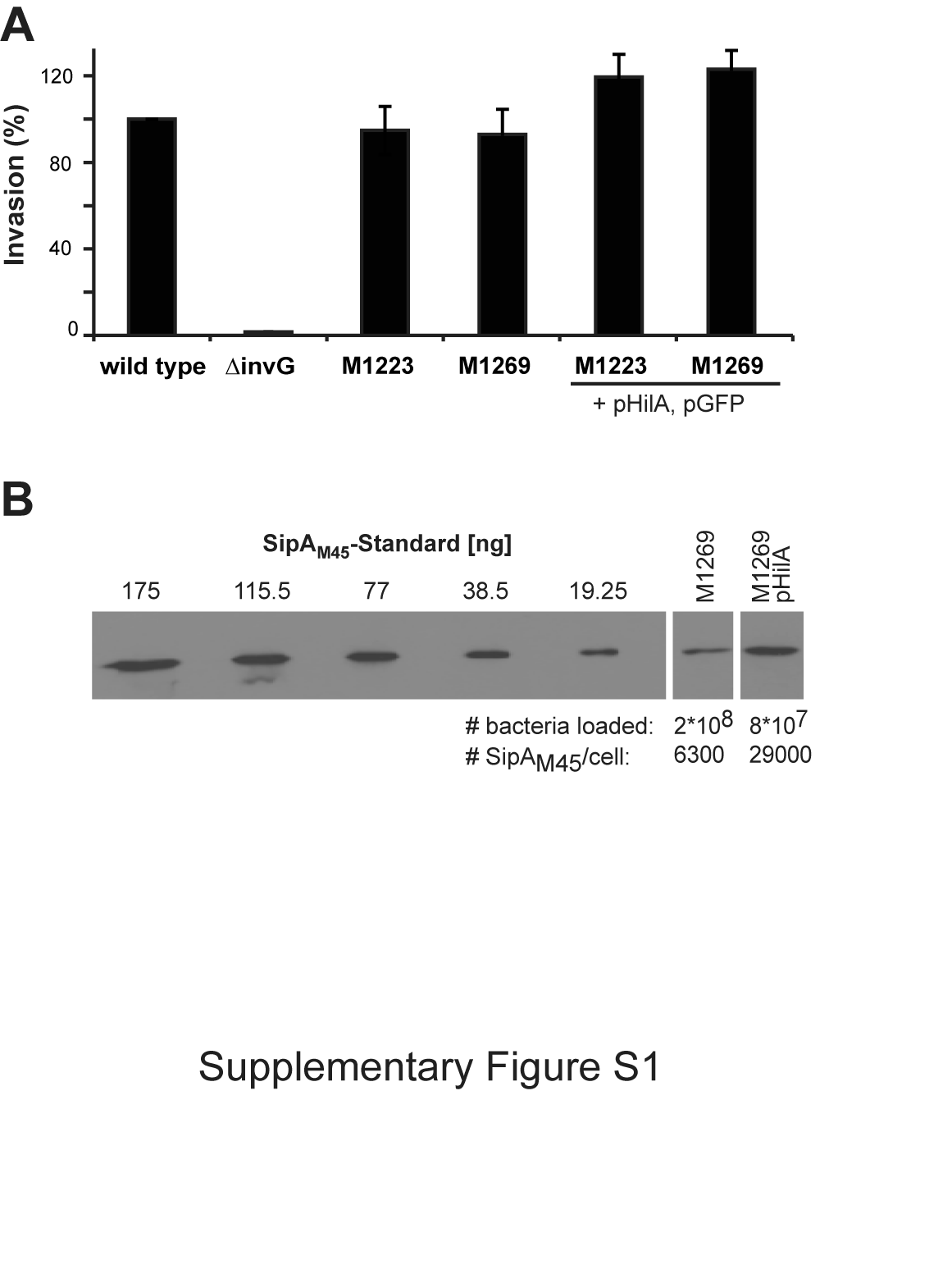

Supplement: Figure S1 — A. Host cell invasion by hilA over-expressing S. Typhimurium strains. COS7 tissue culture cells were infected (moi = 10) for 50 min with the indicated strains and the invasiveness was analyzed in a gentamycin protection assay, as described [3]. The invasiveness was normalized with respect to the number of wild type S. Typhimurium recovered from within the Cos7 cells. A mutant with a disrupted TTSS-1 apparatus (SB161, ΔinvG; [4]) served as a negative control. The data were derived from three independent experiments. They verified that hilA over-expression did not impair TTSS-1 function. B. Typical quantitative Western blot for analyzing the number of effector proteins present per TTSS-1 expressing bacterium. The intensities were scanned and analyzed as described in Materials and Methods. The numbers below the blot indicate the numbers of bacteria of the culture (colony forming units; grown under TTSS-1 inducing conditions) which were loaded onto the respective lane. Data from at least three experiments like this were averaged for each strain and each bacterial protein, analyzed. (1.83 MB TIF) [file pone.0002178.s002.tif]

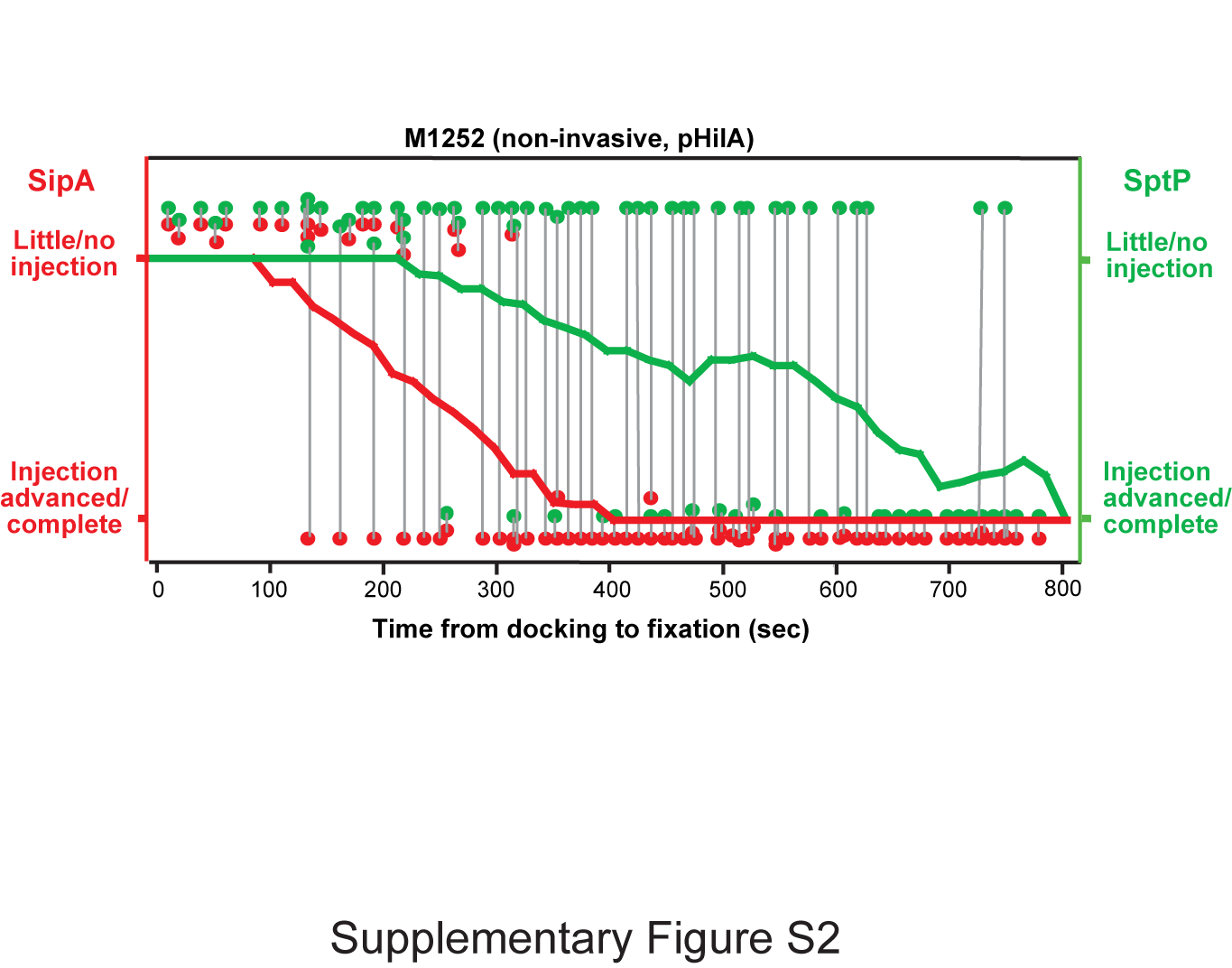

Supplement: Figure S2 — Time course of SipA- and SptP-injection by S. Typhimurium M1252(pHilA). M1252 is an isogenic derivative of M1223 which lacks the key, invasion-mediating effector proteins (sipAM45sptPHA ΔsopABEE2). COS7 cells were infected with M1252(pHilA) and the infection was monitored by time lapse phase contrast microscopy as described in Fig. 2. Cells were fixed, permeabilized with lysozyme, and immuno-stained for LPS (blue), intra-bacterial SipA (red) and intra-bacterial SptP (green). For each bacterium, the graph shows the time between docking and fixation as well as the presence/absence of SipA (red) and SptP (green) in the bacterial cytosol. Gray lines connect SipA and SptP data from the same bacterium. The data was fitted using a rolling average algorithm (red and green lines, see Materials and Methods) to determine when injection was completed with 50% probability (t50%). (4.42 MB TIF) [file pone.0002178.s003.tif]

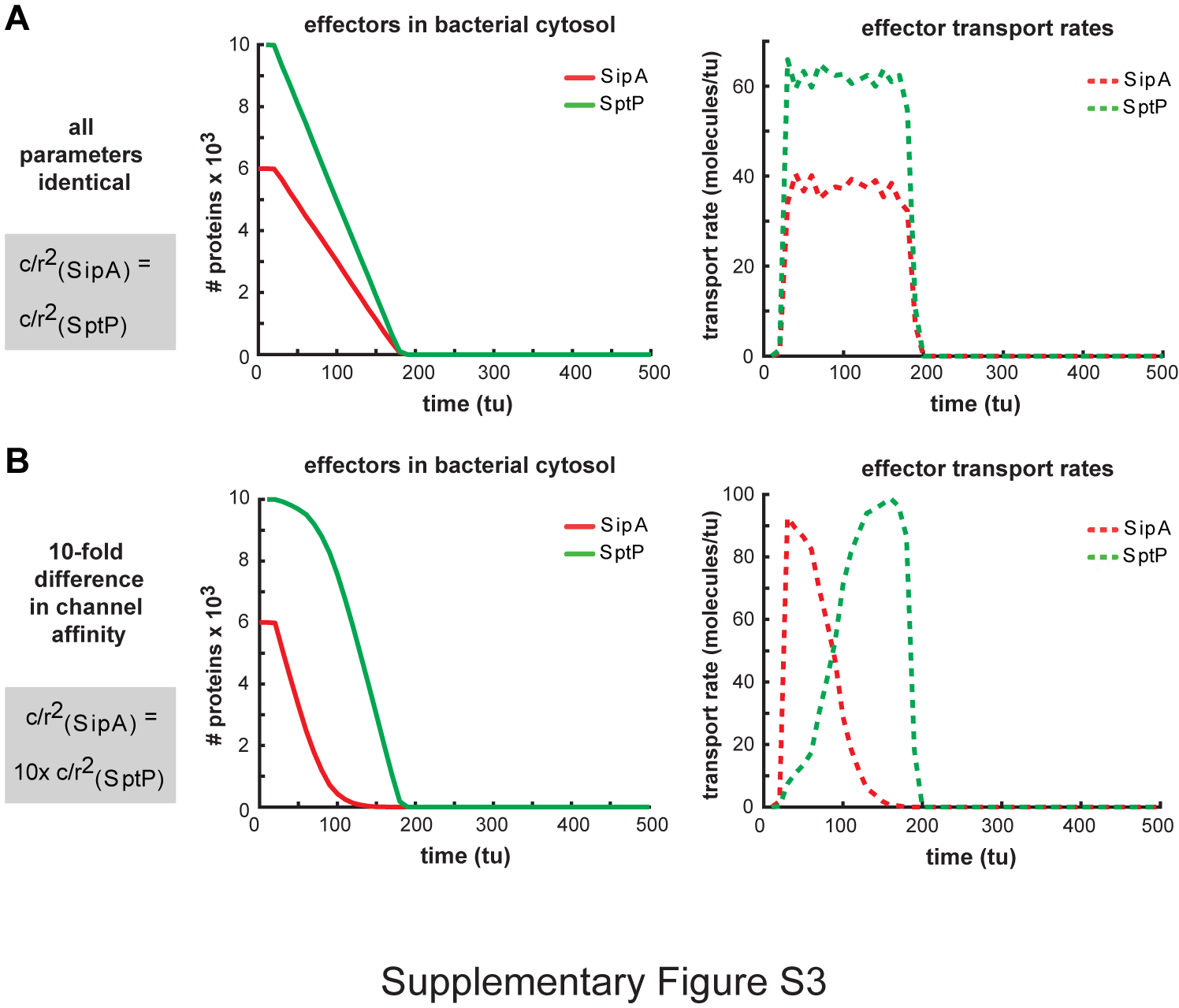

Supplement: Figure S3 — Computer simulation exploring hierarchical SipA and SptP injection by wild type bacteria (e.g. M1269; no hilA over-expression). (A) Simulation of SipA and SptP secretion assuming identical parameters for both effector proteins. Please note that the average “active” wild type S. Typhimurium harbors approx. 10000 molecules of SptP and 6000 molecules of SipA in the cytosol. (B) Simulation of SipA and SptP secretion assuming that SipA-InvB2 has a 10-fold higher affinity (c/r2) for the TTSS than SptP-SicP2. All other steps of SipA- and SptP-secretion had identical parameters. In this case, the bulk of SipA is secreted before SptP. (5.77 MB TIF) [file pone.0002178.s004.tif]

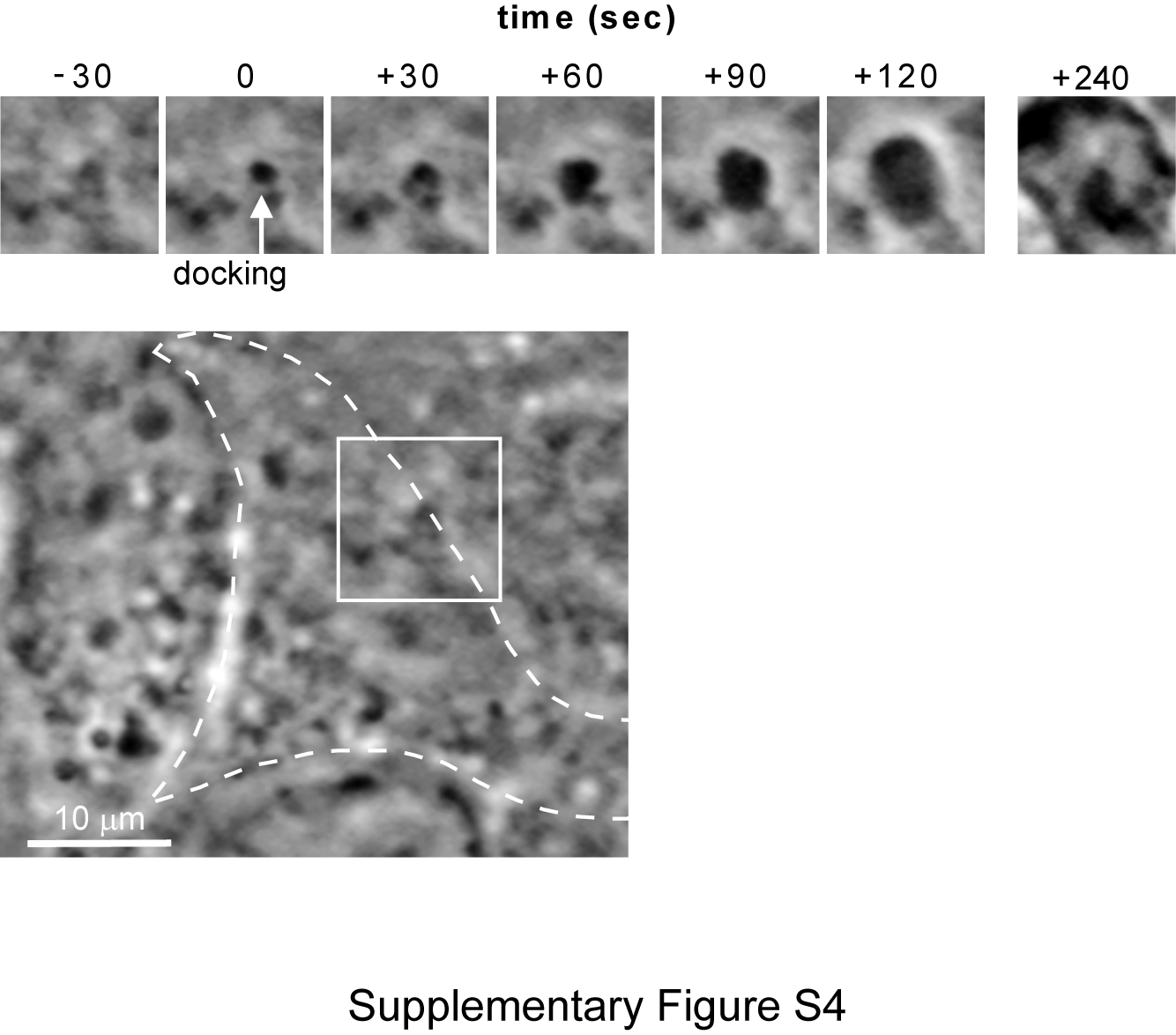

Supplement: Figure S4 — Time course of TTSS-1 induced membrane ruffling. MDCK tissue culture cells were infected with wild type S. Typhimurium. The infection process was monitored on a temperature-controlled stage by phase contrast time lapse microscopy. These data illustrate that membrane ruffling is induced within the first 30–60 seconds after the bacterium has docked to the host cell. The outline of the cell is indicated by the dashed line. (1.59 MB TIF) [file pone.0002178.s005.tif]
